# Interaction of Blood and Bacteria with Slippery Hydrophilic Surfaces

**DOI:** 10.1002/admi.202300564

**Published:** 2023-10-15

**Authors:** Prem Kantam, Vignesh K. Manivasagam, Tarun Kumar Jammu, Roberta Maia Sabino, Sravanthi Vallabhuneni, Young Jae Kim, Arun K. Kota, Ketul C. Popat

**Affiliations:** Department of Mechanical Engineering, Colorado State University, Fort Collins, CO 80523, USA; Department of Mechanical Engineering, Colorado State University, Fort Collins, CO 80523, USA; Department of Mechanical Engineering, Colorado State University, Fort Collins, CO 80523, USA; School of Advanced Materials Discovery, Colorado State University, Fort Collins, CO 80523, USA; Department of Chemical and Biomedical Engineering, University of Wyoming, Laramie, WY 82071, USA; Department of Mechanical and Aerospace Engineering, North Carolina State University, Raleigh, NC 27695, USA; Department of Mechanical and Aerospace Engineering, North Carolina State University, Raleigh, NC 27695, USA; Department of Mechanical Engineering, Colorado State University, Fort Collins, CO 80523, USA; Department of Mechanical and Aerospace Engineering, North Carolina State University, Raleigh, NC 27695, USA; Department of Mechanical Engineering, Colorado State University, Fort Collins, CO 80523, USA; School of Advanced Materials Discovery, Colorado State University, Fort Collins, CO 80523, USA; School of Biomedical Engineering, Colorado State University, Fort Collins, CO 80523, USA

**Keywords:** bacterial adhesion, hydrophilicity, platelet activation, platelet adhesion, slippery

## Abstract

Slippery surfaces (i.e., surfaces that display high liquid droplet mobility) are receiving significant attention due to their biofluidic applications. Non-textured, all-solid, slippery hydrophilic (SLIC) surfaces are an emerging class of rare and counter-intuitive surfaces. In this work, the interactions of blood and bacteria with SLIC surfaces are investigated. The SLIC surfaces demonstrate significantly lower platelet and leukocyte adhesion (≈97.2% decrease in surface coverage), and correspondingly low platelet activation, as well as significantly lower bacterial adhesion (≈99.7% decrease in surface coverage of live *Escherichia Coli* and ≈99.6% decrease in surface coverage of live *Staphylococcus Aureus*) and proliferation compared to untreated silicon substrates, indicating their potential for practical biomedical applications. The study envisions that the SLIC surfaces will pave the path to improved biomedical devices with favorable blood and bacteria interactions.

## 1. Introduction

Slippery surfaces (i.e., surfaces that display high liquid droplet mobility) have received significant attention for their advantages in multiple thermofluidic and biofluidic applications like condensation heat transfer, drag reduction, bio-fouling, miniaturized lab-on-a-chip platforms, etc.^[1]^ Slippery surfaces can be broadly classified as: i) super-repellent surfaces, ii) lubricant-infused surfaces, and iii) non-textured, all-solid surfaces (with covalently attached brushes) based on their underlying mechanism for slipperiness. Typically, super-repellent surfaces and lubricant-infused surfaces utilize textured substrates with lubricant (air or an immiscible liquid) trapped within the texture, inducing slip at the droplet-substrate interface, resulting in high droplet mobility on the surface.^[2]^ Despite the appeal of super-repellent surfaces and lubricant-infused surfaces, they are prone to loss in slipperiness due to texture damage, loss of trapped air due to dissolution over time, depletion of liquid lubricant due to evaporation overtime, etc.^[3]^ On the other hand, slippery non-textured, all-solid surfaces consist of smooth solid substrates with covalently attached oligomeric or polymeric brushes.^[4]^ Slippery non-textured, all-solid surfaces have been receiving increasing attention because they mitigate the common texture-related concerns prevalent in super-repellent surfaces and lubricant-infused surfaces. Among non-textured, all-solid, slippery surfaces, almost all are hydrophobic.^[4,5]^ In our recent work, we elucidated the design of non-textured, all-solid, slippery hydrophilic surfaces^[6]^; such slippery hydrophilic (SLIC) surfaces are counter-intuitive because water droplets spread (rather than bead up) on SLIC surfaces suggesting higher adhesion, and yet water droplets easily slide past SLIC surfaces implying slipperiness. SLIC surfaces constitute an emerging class of surfaces, and their potential biomedical applications are yet to be investigated.

In many biomedical applications, blood-material and bacteria-material interactions play a very important role. Many medical devices, implants, or equipment are prone to failure due to lack of favorable blood-material interactions or due to bacterial biofilm formation. When a medical device, implant, or equipment contacts blood, the initial event that occurs is the adsorption of plasma proteins.^[7]^ This can subsequently trigger the coagulation cascade, which leads to formation of thrombin.^[8]^ Thrombin then plays a key role in converting fibrinogen into a fibrin mesh.^[9]^ The fibrin mesh further instigates platelet and leukocyte adhesion, leading to thrombosis, which disrupts the functionality of the medical device, implant, or equipment.^[10]^ So, surfaces that can reduce thrombosis by inducing favorable blood-material interactions (e.g., reduced platelet and leukocyte adhesion) are highly desirable.^[11]^ Similarly, medical device, implant, or equipment-related infections arise from unfavorable surface interactions with pathogens like bacteria, leading to the formation of biofilms and bacterial colonies, which tend to be resistant to antibiotics.^[12]^ So, surfaces that can reduce biofilm formation by inducing favorable bacteria-material interactions (e.g., reduced adhesion of bacteria) are highly desirable.^[13]^ While blood-material and bacteria-material interactions have been studied for many different kinds of slippery surfaces (e.g., super-repellent surfaces^[8,14]^ or lubricant-infused surfaces^[2d,15]^), there have been no prior investigations of blood-material and bacteria-material interactions on SLIC surfaces.

In our recent work, we observed that SLIC surfaces can significantly reduce fibrinogen adsorption (i.e., one of the first steps in the blood-coagulation cascade) compared to non-slippery hydrophilic surfaces and slippery hydrophobic surfaces.^[6]^ Building on this promising observation, in this study, we investigated the interactions of blood components (platelets and leukocytes) and bacteria (gram-positive or gram-negative) with SLIC surfaces. Based on the results in this study, SLIC surfaces demonstrated ≈97.2% lower surface coverage (i.e., nearly two-log reduction) in platelet and leukocyte adhesion, and correspondingly lower platelet activation, as well as significantly lower bacterial adhesion (≈ 99.7% decrease in surface coverage of live *Escherichia Coli (E. coli)* and ≈99.6% decrease in surface coverage of live *Staphylococcus Aureus (S. aureus)*, i.e., nearly two-log reduction) compared to untreated silicon substrates, indicating their potential for practical biomedical applications. We envision that our SLIC surfaces will pave the path to improved biomedical devices with reduced blood and bacteria interactions.

## 2. Results and Discussion

SLIC surfaces display both hydrophilicity (low contact angle with water) and slipperiness (high liquid droplet mobility) simultaneously. We fabricated SLIC surfaces by covalently grafting high surface energy polyethylene glycol (PEG) brushes to smooth solid substrates (silicon wafers) via liquid phase silanization (see Experimental Section).^[6]^ The high surface energy PEG brushes imparted hydrophilicity to the SLIC surfaces. The low physical inhomogeneity (due to low surface roughness) and low chemical inhomogeneity (due to sufficiently high grafting density) resulted in low contact angle hysteresis ($\Delta \theta =\theta_{\text{adv}}-\theta_{\text{rec}}$, where $\theta_{\text{adv}}$ is the advancing contact angle and $\theta_{\text{rec}}$ is the receding contact angle), which in turn led to slipperiness.

**Figure 1.**
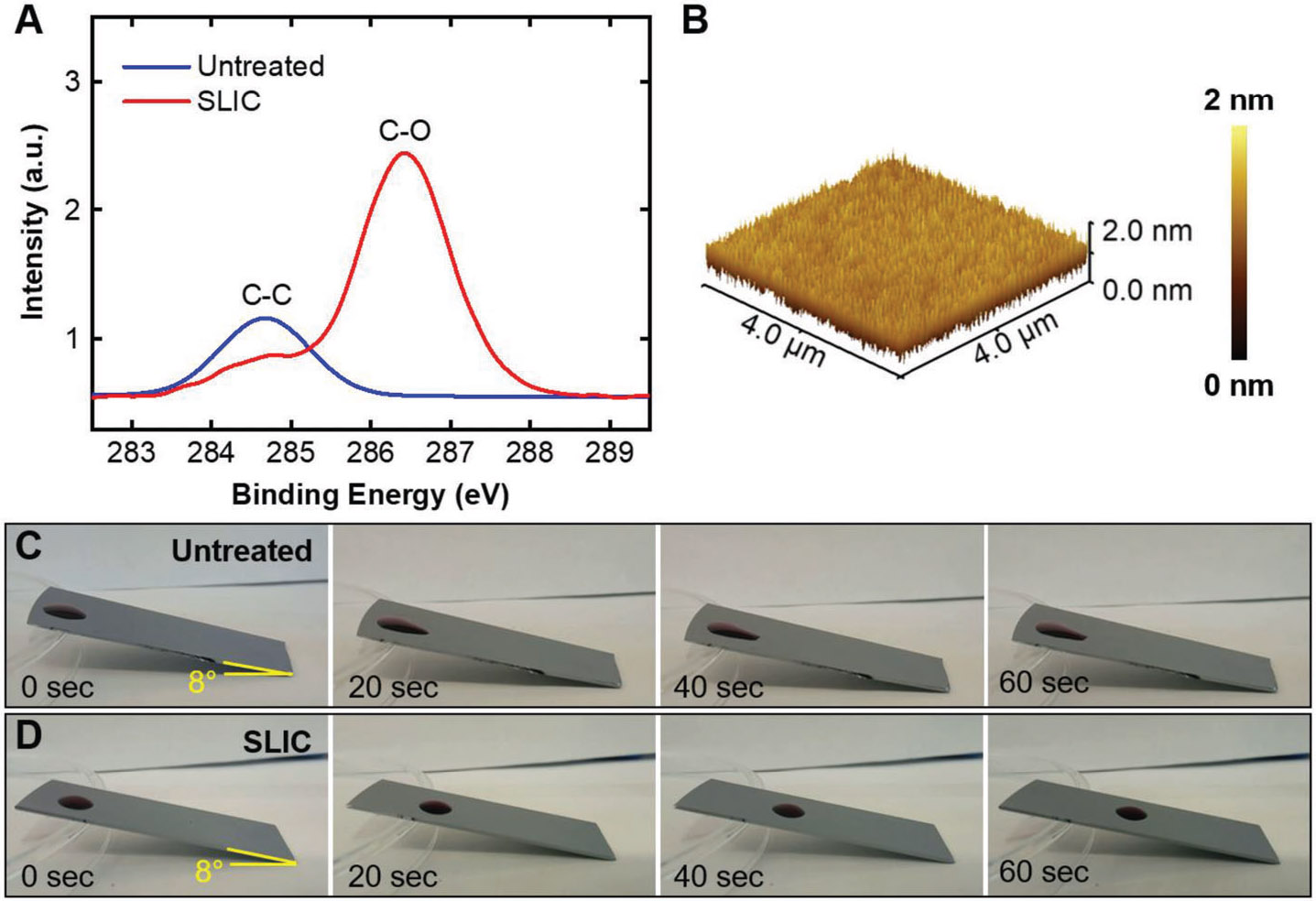
Characterization of SLIC surfaces. A) High-resolution C1s XPS spectra of untreated silicon and SLIC surfaces. The C─C peak on untreated silicon is due to the presence of adventitious carbon. The C─O peak on SLIC surface indicates the presence of PEG functional groups. B) AFM image depicting the topography of SLIC surface with low surface roughness ($R_{\mathrm{rms}}<1\;\mathrm{nm}$). C) Time-lapse images of a 20 μL droplet adhered (i.e., not sliding) on an untreated silicon surface at a tilt angle of 8°. D) Time-lapse images of a 20 μL droplet sliding on a SLIC surface at a tilt angle of 8°.

We characterized the surface chemistry (i.e., functional group), surface roughness, and surface wettability (i.e., hydrophilicity and slipperiness) of our SLIC surfaces using x-ray photoelectron spectroscopy (XPS), atomic force microscopy (AFM) and contact angle goniometry, respectively (see Experimental Section). The presence of C─O peak at 286.5 eV on high-resolution C1s XPS spectrum indicated the presence of PEG functional groups on our SLIC surfaces (see Figure 1A; Section S1, Supporting Information). The AFM images of our SLIC surfaces indicated a low surface roughness ($R_{\text{rms}}<1\;\text{nm}$; see Figure 1B). Our SLIC surfaces displayed hydrophilicity with $\theta_{\text{adv}}\approx 41^{{}^{\circ}}$ and $\theta_{\text{rec}}\approx 37^{{}^{\circ}}$ and slipperiness with low contact angle hysteresis $\Delta \theta \approx 4^{{}^{\circ}}$ (also see Section S2, Supporting Information). The slipperiness of SLIC surfaces was also evident from 20 μL water droplets sliding past the surface at a tilt angle of (8°, while 20 μL water droplets did not slide past an untreated silicon wafer at the same tilt angle (see Figure 1C,D). Our SLIC surfaces retained their hydrophilicity and slipperiness for at least 5 days upon exposure to air and steam, immersion under water, and for at least 50 000 water droplets sliding past the surface (see Section S3, Supporting Information).

**Figure 2.**
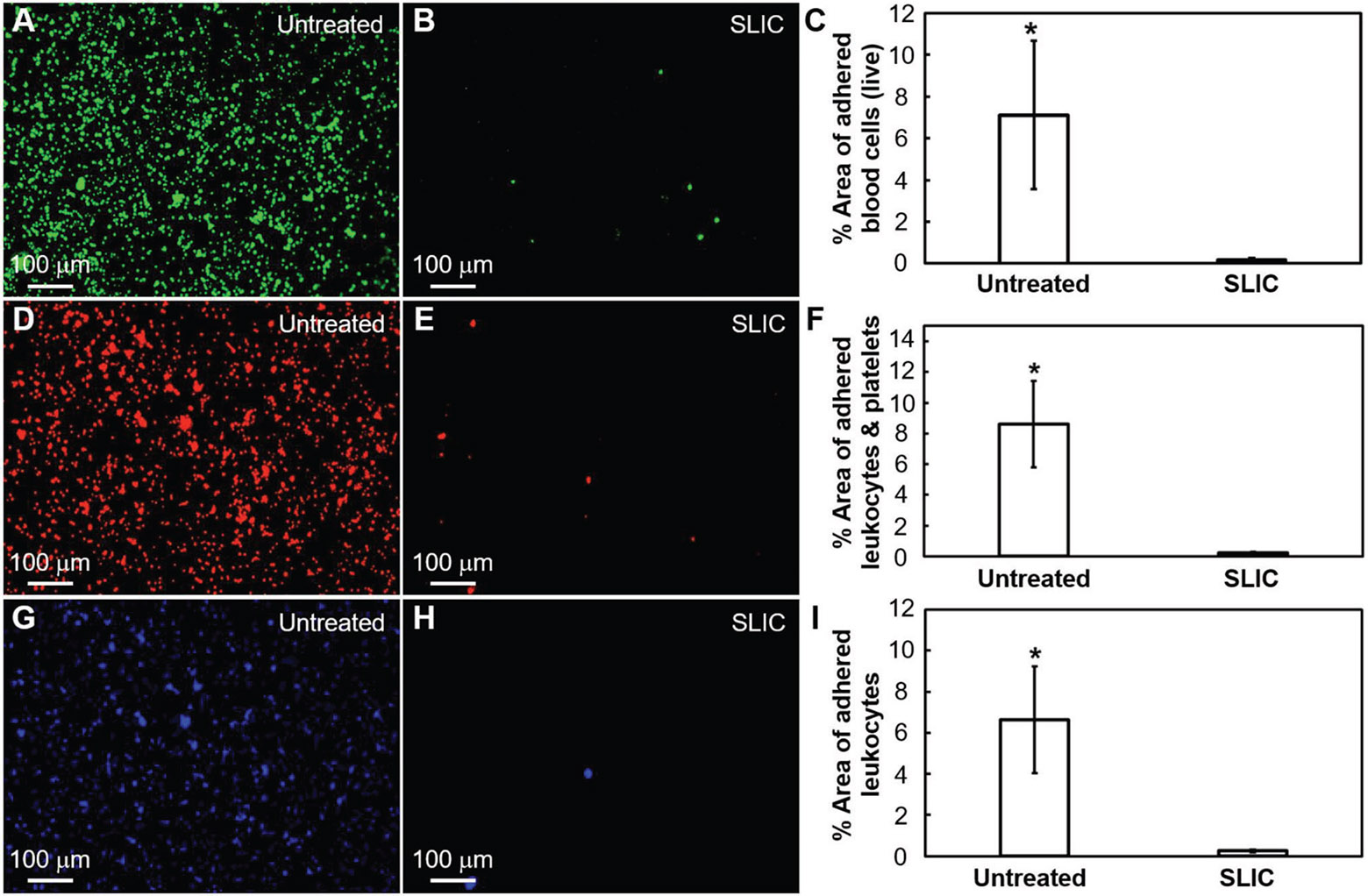
Platelet & leukocyte adhesion on SLIC surfaces. A,B) Fluorescent microscopy images showing blood cell (live) adhesion (stained green) on untreated silicon and SLIC surfaces, respectively. C) Bar chart indicating significant surface coverage (*indicates *p* < 0.05) of live blood cells on untreated silicon and SLIC surfaces. D,E) Fluorescent microscopy images showing platelet & leukocyte adhesion (stained red) on untreated silicon and SLIC surfaces, respectively. F) Bar chart indicating significant surface coverage (*indicates *p* < 0.05) of platelets & leukocytes (combined) on untreated silicon and SLIC surfaces. G,H) Fluorescent microscopy images showing leukocyte adhesion (stained blue) on untreated silicon and SLIC surfaces, respectively. I) Bar chart indicating significant surface coverage (*indicates *p* < 0.05) of leukocytes on untreated silicon and SLIC surfaces.

**Figure 3.**
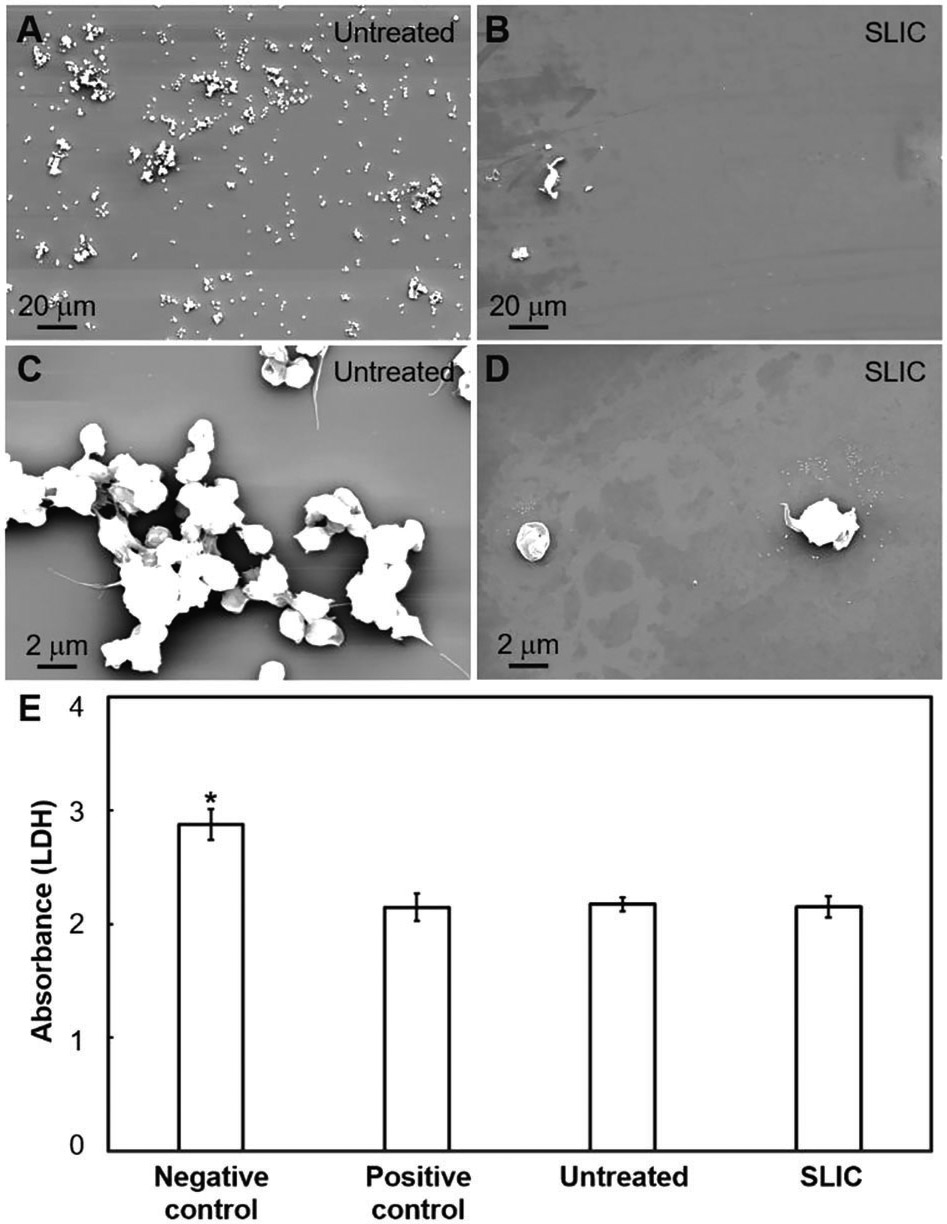
Platelet activation and cytotoxicity on SLIC surfaces. A,B) SEM images showing platelet activation on untreated silicon and SLIC surfaces, respectively at lower magnification. C,D) SEM images showing platelet activation on untreated silicon and SLIC surfaces, respectively at higher magnification. E) Bar chart comparing the cytotoxicity on negative control, positive control, untreated silicon and SLIC surfaces (*indicates *p* < 0.05).

We investigated the interaction of blood platelets and leukocytes with our SLIC surfaces using platelet-rich plasma (PRP) obtained from human blood (see Experimental Section). When blood contacts a foreign solid surface, fibrinogen (a blood protein) adsorbs on the surface.^[10,16]^ This further activates the aggregation of platelets and leukocytes that are present in blood, resulting in blood coagulation.^[16,17]^ In our prior work, we demonstrated significantly delayed fibrinogen adsorption (i.e., one of the first steps in the blood-coagulation cascade) on SLIC surfaces, which could possibly hinder the subsequent steps in the blood-coagulation cascade.^[1a,16,18]^ To further investigate this, we studied platelet and leukocyte adhesion and platelet activation on our SLIC surfaces and compared it with untreated silicon surfaces. We exposed untreated silicon surfaces and SLIC surfaces to PRP for 2 h and characterized the platelet and leukocyte adhesion using fluorescence microscopy after staining live blood cells (i.e., platelets & leukocytes) with calcein-AM (see Experimental Section). We quantified the adhesion of platelets and leukocytes by determining the surface coverage on our substrates using the images acquired from fluorescence microscopy. After 2 h exposure to PRP, we observed a significantly lower adhesion of platelets and leukocytes on SLIC surfaces compared to untreated surfaces (see Figure 2A-I). Our results indicate that there is a 97.5% decrease in the surface coverage of live blood cells on SLIC surfaces (see Figure 2A-C). In addition, to identify the independent adhesion of platelets and leukocytes, we used fluorescence microscopy to investigate the adhesion of fixed blood cells after staining them with rhodamine-phalloidin (actin) and 4, 6-diamidino-2-phenylindole (DAPI). Actin stains the cytoskeleton of both platelets and leukocytes red, while DAPI stains only the nucleus of leukocytes blue. Our results indicate that there is a 97.2% decrease in adhered platelets and leukocytes (combined; see Figure 2D-F) and 96.2% decrease in the adhered leukocytes (only; see Figure 2G-I) on SLIC surfaces. We also characterized the platelet activation on our surfaces using SEM (see Experimental section) and observed a significantly lower activation of platelets on SLIC surfaces compared to untreated surfaces (see Figure 3A-D).

We also characterized the cytotoxicity on our surfaces to ensure that SLIC surfaces are not toxic to the contacting blood cells and can enable safe interactions with blood. We evaluated cytotoxicity using lactate dehydrogenase (LDH) assay (see Experimental Section) and compared the cytotoxicity on untreated surfaces, SLIC surfaces, positive control (with 100% live cells), and negative control (with 100% dead cells). Typically, blood cells produce LDH enzyme due to loss of membrane integrity during their death.^[19]^ Thus, the presence of LDH enzyme in blood can be correlated with cytotoxicity on the surface.^[19a,20]^ Our results indicate that the cytotoxicity on SLIC surfaces are not significantly different from that on untreated surfaces and positive control (see Figure 3E). However, the negative control demonstrated significantly higher cytotoxicity due to the presence of dead cells. This further confirms that SLIC surfaces can enable safe interactions with blood cells without leading to their death.

**Figure 4.**
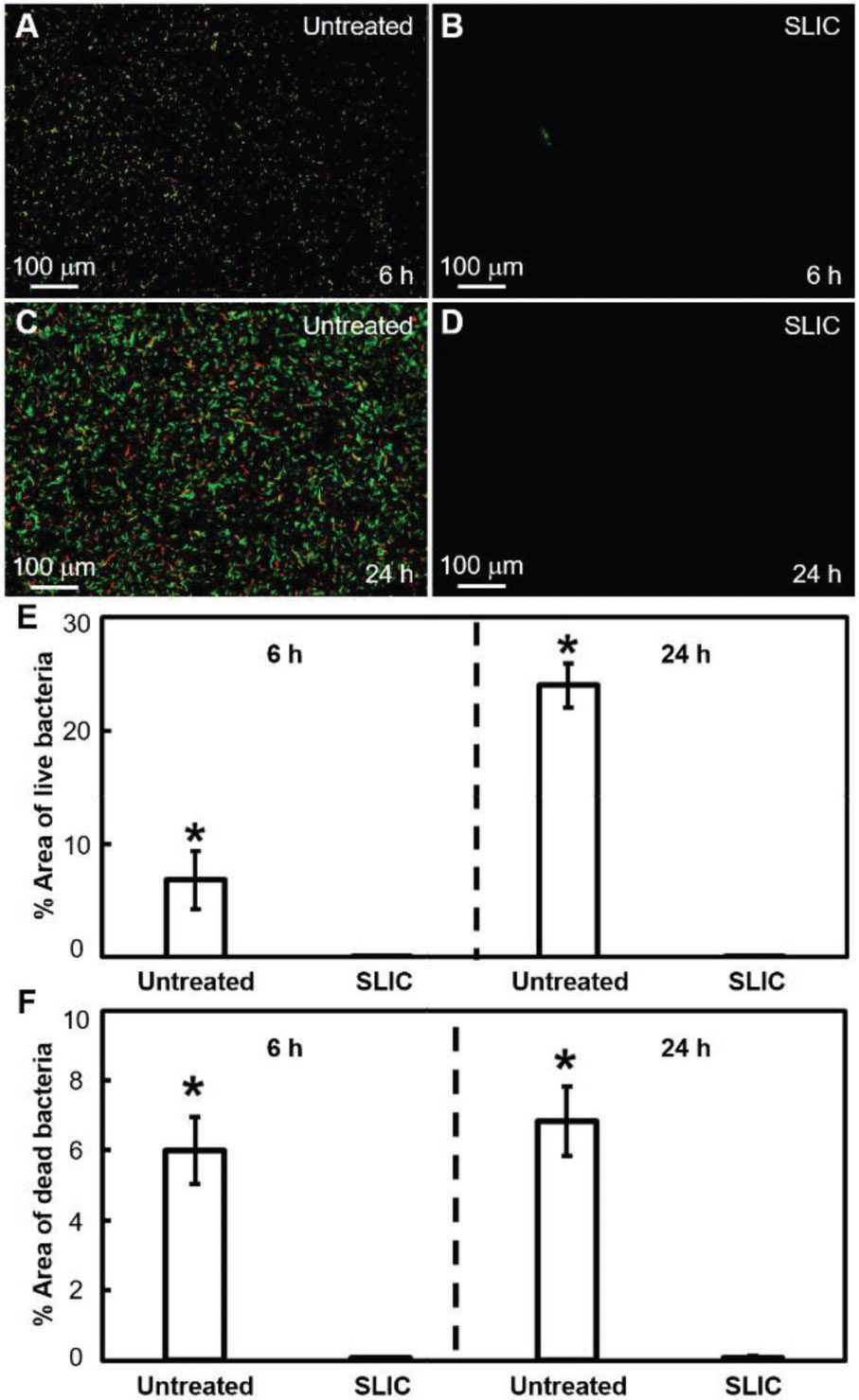
*E. coli* adhesion on SLIC surfaces using fluorescence microscopy. A,B) Fluorescent microscopy images showing *E. coli* adhesion on untreated silicon and SLIC surfaces, respectively after 6 h of incubation. C,D) Fluorescent microscopy images showing *E. coli* adhesion on untreated silicon and SLIC surfaces, respectively after 24 h of incubation. E,F) Bar charts indicating the significant surface coverage (*indicates *p* < 0.05) of live and dead *E. coli*, respectively on untreated silicon compared to SLIC surfaces at 6 and 24 h of incubation. The area fraction of bacteria adhesion was ≈0.1% on our SLIC surfaces, and there was no discernible change at 24 h compared to 6 h.

**Figure 5.**
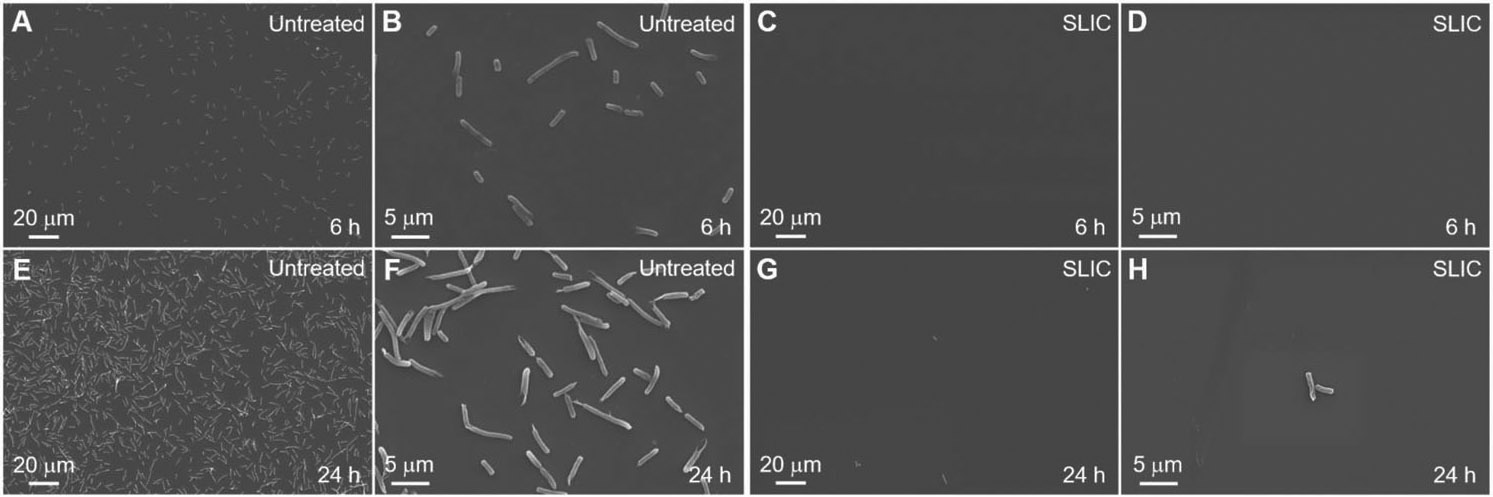
*E. coli* adhesion on SLIC surfaces using SEM. A–D) SEM images showing *E. coli* adhesion on untreated silicon and SLIC surfaces, respectively after 6 h of incubation. E–H) SEM images showing *E. coli* adhesion on untreated silicon and SLIC surfaces, respectively after 24 h of incubation.

**Figure 6.**
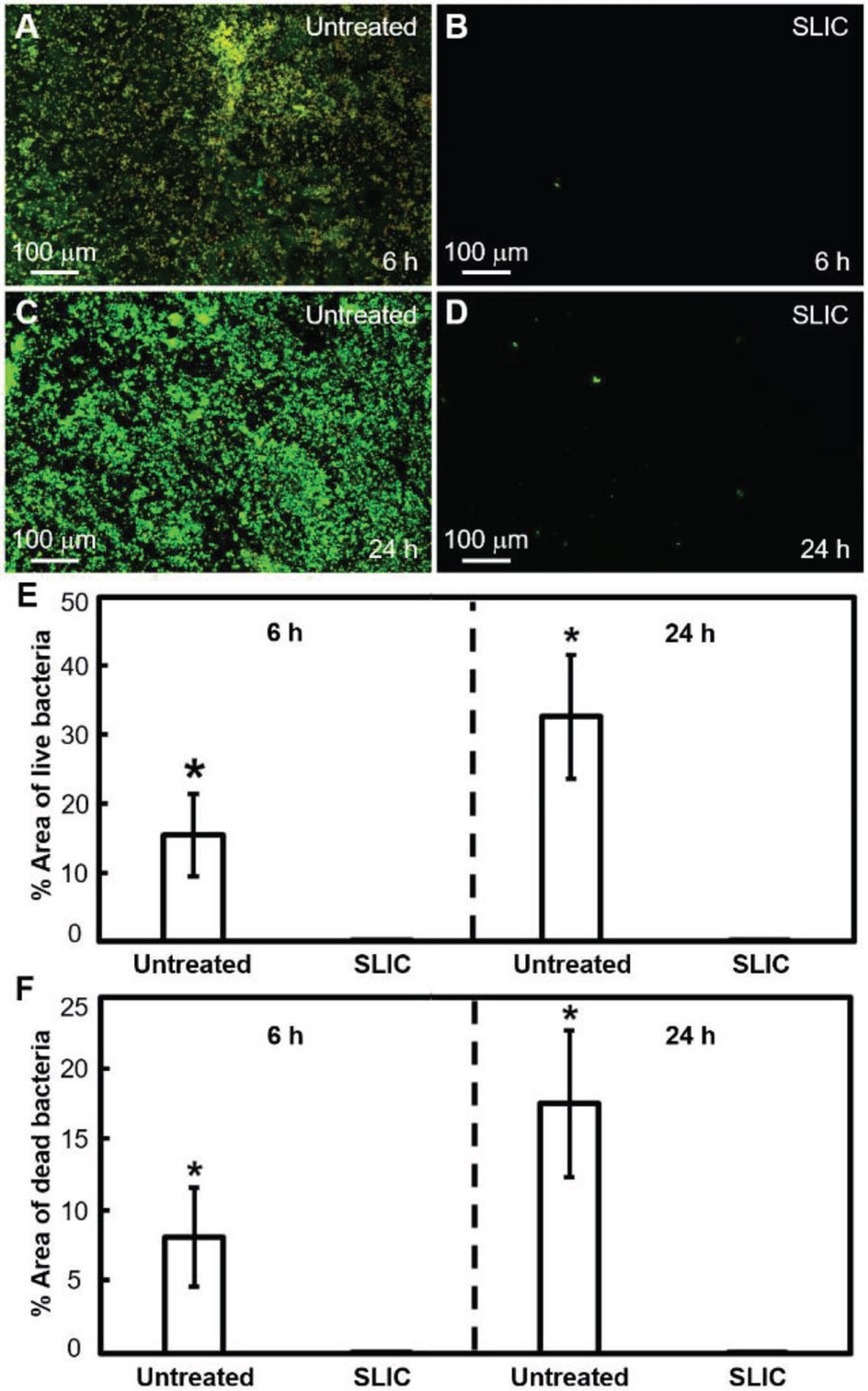
*S. aureus* adhesion on SLIC surfaces using fluorescence microscopy. A,B) Fluorescent microscopy images showing *S. aureus* adhesion on untreated silicon and SLIC surfaces, respectively after 6 h of incubation. C,D) Fluorescent microscopy images showing *S. aureus* adhesion on untreated silicon and SLIC surfaces, respectively after 24 h of incubation. E,F) Bar charts indicating the significant surface coverage (*indicates *p* < 0.05) of live and dead *S. aureus*, respectively on untreated silicon compared to SLIC surfaces at 6 and 24 h of incubation. The area fraction of bacteria adhesion was ≈0.1% on our SLIC surfaces, and there was no discernible change at 24 h compared to 6 h.

**Figure 7.**
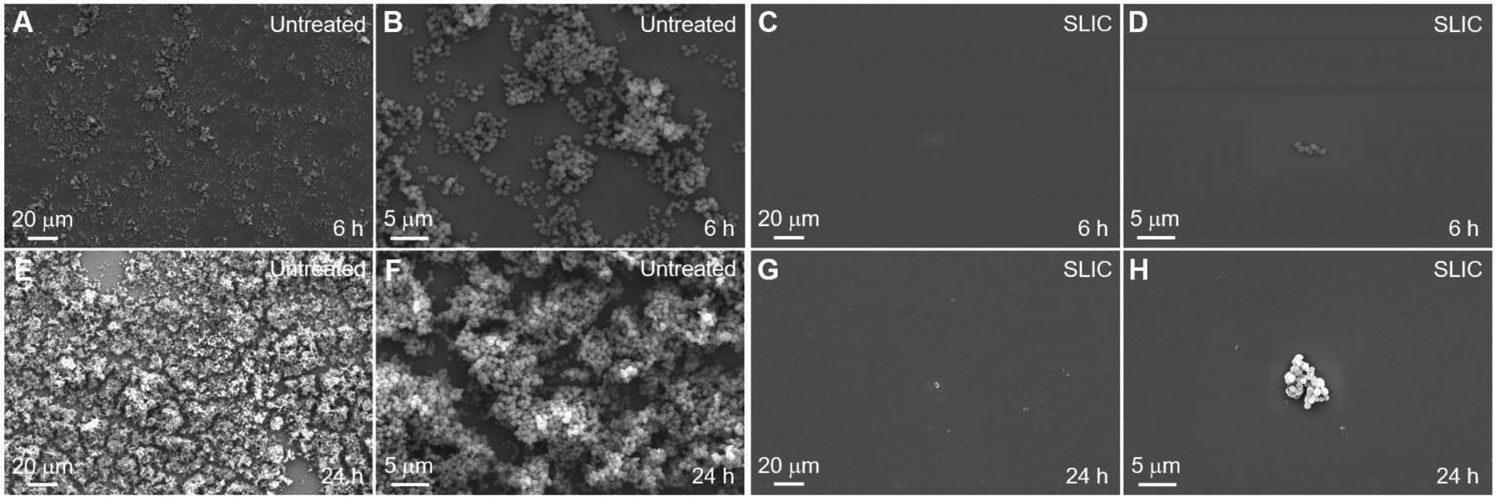
*S. aureus* adhesion on SLIC surfaces using SEM. A–D) SEM images showing *S. aureus* adhesion on untreated silicon and SLIC surfaces, respectively after 6 h of incubation. E–H) SEM images showing *S. aureus* adhesion on untreated silicon and SLIC surfaces, respectively after 24 h of incubation.

In addition to studying blood interactions, we investigated bacterial activity on our SLIC surfaces to demonstrate their potential for blood-contacting biomedical devices. In biomedical devices (e.g., body implants, catheters, etc.), bacterial infections can arise due to the adsorption of bacteria from contamination of biomedical devices, bacteria inside or on the patient’s body, contamination of surgical equipment, etc.^[21]^ Furthermore, adsorbed bacteria on a surface promote the growth of bacterial colonies leading to the formation of biofilms.^[21b,22]^ These biofilms contain proteins and polysaccharides, which shield the bacteria from antibiotics administered to the patient.^[21b,23]^ So, it is important to investigate the interaction of bacteria with our SLIC surfaces to better understand their functionality, risk of infection, and failure. To investigate this, we studied the interaction of *E. coli* (i.e., a gram-negative bacteria) and *S. aureus* (i.e., a gram-positive bacteria) with our SLIC surfaces. We chose *E. coli* because it has a high propensity to cause biomedical device-related infections,^[22]^ and we chose *S. aureus* because it has a high propensity to form biofilms with extraordinary resistance to both antibiotics and immune responses.^[24]^ We characterized the interaction of bacteria with the surfaces using fluorescence microscopy after staining the live and dead bacteria with propidium iodide, and SEM (see Experimental section). Propidium iodide stains the live bacteria green and the dead bacteria red. We evaluated the interaction of bacteria with SLIC surfaces by incubating them in bacterial solution for 6 and 24 h (see Experimental section) and comparing the bacterial adhesion and proliferation with that on untreated surfaces. Our fluorescent images indicate a significant decrease in bacterial adhesion and proliferation (with both *E. coli* and *S. aureus*), at both 6 and 24 h incubation times, on SLIC surfaces compared to those on untreated surfaces (see Figures 4-7). Specifically, our fluorescent images showed a 98.6% decrease in live *E. coli*, and 98.8% decrease in dead *E. coli* after 6 h of incubation, and 99.7% decrease in live *E. coli* and 98.9% decrease in dead *E. coli* after 24 h of incubation on SLIC surfaces compared to untreated surfaces (see Figure 4A-F). Correspondingly, our SEM images also indicated a significant decrease in adhesion and proliferation of *E. coli* on SLIC surfaces compared to untreated surfaces (see Figure 5). Our fluorescent images also indicated 99.6% decrease in live *S. aureus* and 98.8% decrease in dead *S. aureus* after 6 h of incubation, and 99.6% decrease in live *S. aureus* and 99.7% decrease in dead *S. aureus* after 24 h of incubation on SLIC surfaces compared to untreated surfaces (see Figure 6A-F). Correspondingly, our SEM images also indicated a significant decrease in adhesion and proliferation of *S. aureus* on SLIC surfaces compared to untreated surfaces (see Figure 7). These results demonstrate the potential of our SLIC surfaces in minimizing the interaction of bacteria with the substrate and delaying bacterial adhesion and growth.

## 3. Conclusion

In this work, we investigated the interaction of blood and bacteria on slippery hydrophilic (SLIC) surfaces. We fabricated slippery hydrophilic surfaces by grafting PEG brushes to smooth solid substrates. We investigated the interaction of blood by studying platelet and leukocyte adhesion, and platelet activation. Our results indicate a nearly two-log reduction in platelet and leukocyte adhesion as well as platelet activation on SLIC surfaces compared to untreated silicon surfaces. We investigated the interaction of bacteria by studying the adhesion of *E. coli* and *S. aureus*. Our results indicate a nearly two-log reduction in the adhesion of *E. coli* and *S. aureus* on SLIC surfaces compared to untreated silicon surfaces, even after 24 h incubation. The fouling resistance of our SLIC surfaces is possibly due to strongly bound and highly ordered (“ice-like”) hydration layers,^[25]^ which have the potential to act as a strong barrier to protein adsorption, thereby greatly enhancing fouling resistance.^[25,26]^ A comprehensive investigation of SLIC surfaces, including the role of ordered water molecules in the hydration layers, as well as their durability and applicability to different substrates, is necessary for a complete understanding.

## 4. Experimental Section

### Fabrication of SLIC Surfaces:

Commercially available single side polished silicon wafers with a thickness of 650 μm were cut into 5 cm x 3 cm pieces and cleaned thoroughly via ultrasonication for 10 min with acetone, ethanol, and DI water sequentially. Subsequently, these surfaces were dried using nitrogen. The pre-cleaned wafers were exposed to oxygen plasma (Plasma Etch PE-25) for 15 min for hydroxylation. The hydroxylated silicon wafer surfaces were immersed in a solution of PEG silane (2–methoxy polyenthleneoxy (6-9) propyl trimethoxysilane (Gelest); 3 μL), hydrochloric acid (12 μL), and toluene (40 mL) for 8 h at room temperature. Finally, the silanized silicon substrates were cleaned by rinsing thoroughly with DI water and dried with nitrogen. This simple and scalable liquid-phase silanization-based technique of fabricating SLIC surfaces could be extended to other substrates with sufficiently high hydroxyl groups.

### Characterization of Surface Wettability and Surface Chemistry:

The surface wettability of substrates was characterized using contact angle goniometry. Contact angles (advancing contact angle $\theta_{\text{adv}}$, receding contact angle $\theta_{\text{rec}}$) and contact angle hysteresis CAH were measured using Ramé-Hart 260F4 goniometer. At least three measurements were performed on each substrate at spatially different locations and the error in all the reported data was ±2°.

The surface chemistry on substrates was characterized using XPS (Physical Electronics PHI-5800 spectrophotometer). XPS was conducted using a monochromatic Al Ka X-ray source operated at 15 kV, and photoelectrons were collected at a takeoff angle of 45° relative to the sample surface. XPS data was acquired from at least three spatially different locations on each surface, and the spectral analysis was conducted using PHI Multipak software.

### Isolation of Platelet Rich Plasma (PRP) from Whole Blood:

Whole blood was acquired from healthy donors through venipuncture. The protocol was approved by the Colorado State University Institutional Review Board and was conducted in compliance with the National Institute of Health’s “Guiding Principle for Ethical Research”. Informed consents were obtained from human participants prior to enrolling in the study. Six milliliter tubes coated with the anticoagulant, ethylenediaminetetraacetic acid (EDTA) were used for collecting the blood. The first tube of blood was disposed off to avoid the platelet plug and locally activated platelets from the needle insertion. The PRP was isolated from the blood by centrifuging the tubes at 150 g for 15 min. The centrifuged blood tubes were left idle for 15 min before using the PRP for cell studies on the substrates. For all the biological studies, PRP was pooled from the same donor to account for donor-to-donor variability in the platelet count.

### Characterization of Platelet and Leukocyte Adhesion:

The adhesion of platelets and leukocytes on the substrates was characterized using fluorescence microscopy by staining live blood cells with Calcein-AM stain. All substrates were incubated in a 48-well plate with PRP (400 μL) at 100 rpm on a horizontal shaker for 2 h at 37 °C and 5% CO_2_ (similar to human physiological conditions). PRP was aspirated after incubation and the substrates were rinsed three times with PBS to remove any nonadherent blood cells. These substrates were then stained using 5% Calcein-AM solution in PBS (300 μL) for 20 min at ambient temperature in a dark room. The stain solution was then aspirated, and the surfaces were rinsed two times with PBS to remove any excess stain solution. Finally, the substrates were imaged using a fluorescence microscope (Zeiss AxioVision). The acquired fluorescent images were further processed using ImageJ software to determine the surface coverage of live blood cells on the substrates.

### Identification of Adhered Platelets and Leukocytes on Substrates:

Platelets and leukocytes on the substrates were identified using fluorescence microscopy by staining the adhered blood cells using 4,6–diamidino–2–phenylindole (DAPI) and rhodamine-phalloidin (actin). DAPI stains the nucleus of leukocytes blue while actin stains the cytoskeleton of both platelets and leukocytes red. The substrates were incubated in a 48-well plate with PRP (400 μL) at 100 rpm on a horizontal shaker for 2 h at 37 °C and 5% CO_2_ (similar to human physiological conditions). PRP was aspirated after incubation and the substrates were rinsed three times with PBS to remove any nonadherent blood cells. Subsequently, the substrates were fixed by incubating them in 3.7% formaldehyde solution for 15 min. Later, the substrates were rinsed three times with PBS and incubated in a solution of 1% Triton X for 3 min. The substrates were further rinsed three times with PBS and transferred to another 48-well plate. Subsequently, the substrates were incubated in 0.05% actin solution (300 μL) for 20 min and were incubated in 3% DAPI stain stock solution (21 μL) for 5 min. The substrates were then rinsed two times with PBS and imaged using a fluorescence microscope. All acquired fluorescent images were processed using ImageJ software to determine actin cell coverage and number of nuclei adhered to the substrates.

### Characterization of Platelet Activation:

Platelet activation on the substrates was characterized using SEM. All substrates were incubated in a 48-well plate with PRP (400 μL) at 100 rpm on a horizontal shaker for 2 h at 37 °C and 5% CO_2_. PRP was aspirated after incubation and the substrates were rinsed three times with PBS to remove any nonadherent cells. The adhered cells were fixed by incubating the substrates in a solution containing 6% glutaraldehyde, 0.1 m sodium cacodylate, and 0.1 m sucrose in DI water for 45 min. The substrates were then transferred to a buffer solution containing 0.1 m sodium cacodylate and 0.1 m sucrose for 10 min. Subsequently, the substrates were sequentially incubated in 35%, 50%, 70%, and 100% ethanol solutions for 10 min each. Finally, the substrates were air-dried and imaged using an SEM (JOEL 6500 field emission scanning electron microscope). All the substrates were sputter coated with 5 nm of gold and the SEM images were acquired at an accelerating voltage of 15 kV.

### Culturing Bacteria for Bacterial Adhesion Study:

*Escherichia Coli* (i.e., gram-negative bacteria) and *Staphylococcus Aureus* (i.e., gram-positive bacteria) were utilized to investigate bacterial activity on the substrates. Bacteria was incubated in a tryptic soy broth (TSB) medium for 8 h at 37 °C (similar to human physiological conditions). The initial concentrations of the bacteria solution were determined using a plate reader at a wavelength of 562 nm. The bacteria solution was diluted for the absorbance value of 0.52 to represent a bacteria concentration of 10^9^ (CFU mL^−1^). Subsequently, the bacteria solution was diluted to 10^6^ (CFU mL^−1^) for the study. Finally, the substrates were incubated in bacterial solution (500 μL) for 6 and 24 h at 37 °C to characterize bacterial activity.

### Characterization of Bacterial Activity:

Bacterial activity on the substrates was investigated by characterizing bacterial adhesion and bacteria colonization. Bacterial adhesion was characterized using fluorescence microscopy. After incubation in bacterial solution, the substrates were stained with live/dead bacteria stain solution (500 μL) by combining equal amounts of 20 mm propidium iodide and 3.34 mm Syto 9 for 15 min at room temperature. The substrates were then rinsed with PBS (500 μL) for 5 min. The adhered bacteria on the substrates were fixed by incubating them in 3.7% formaldehyde solution for 15 min at room temperature. The formaldehyde solution was aspirated, and the substrates were rinsed twice with PBS before imaging with a fluorescence microscope. All acquired fluorescence images were processed using ImageJ software to determine the surface coverage of live/dead bacteria.

Bacteria proliferation on the substrates was characterized using SEM. After incubation in bacterial solution, the adhered bacteria on the substrates were fixed by incubation in a primary fixative solution containing 3.7% glutaraldehyde, 0.1 m sodium cacodylate, and 0.1 m sucrose in DI water for 45 min. Substrates were then transferred to a buffer solution containing 0.1 m sodium cacodylate and 0.1 m sucrose for 10 min. Subsequently, the substrates were sequentially incubated in 35%, 50%, 70%, and 100% ethanol solutions for 10 min each. Finally, they were air-dried and imaged using an SEM (JOEL 6500 field emission scanning electron microscope). Before imaging, all substrates were sputter-coated with 5 nm of gold.

### Statistical Analysis:

Contact angle and sliding angle measurements, as well as fluorescence imaging, were conducted and reported based on at least three samples for each type of substrate at three spatially different locations on each sample ($n_{\min}=9$). The cytotoxicity experiment was carried out with $n_{\min}=4$. SEM imaging and XPS analysis were conducted on at least three different samples ($n_{\min}=3$) for each type of substrate. All data were presented as mean ± SD unless otherwise specified. All quantitative results were analyzed statistically using JMP Pro software and the results were considered statistically significant if *p*-value <0.05.

## Supplementary Material

SI Supporting Information is available from the Wiley Online Library or from the author.

## Data Availability

The data that support the findings of this study are available from the corresponding author upon reasonable request.
