# Supplementary material for: Interaction of Blood and Bacteria with Slippery Hydrophilic Surfaces
Source: Adv Mater Interfaces. Author manuscript; Available in PMC 2025 Jun 12. (PMC12162091; doi:10.1002/admi.202300564)
Supplement: SI [file NIHMS2086463-supplement-SI.pdf]

ADVANCED MATERIALS INTERFACES
Open Access

Supporting Information

for *Adv. Mater. Interfaces*, DOI 10.1002/admi.202300564

Interaction of Blood and Bacteria with Slippery Hydrophilic Surfaces

Prem Kantam, Vignesh K. Manivasagam, Tarun Kumar Jammu, Roberta Maia Sabino, Sravanthi Vallabhuneni, Young Jae Kim, Arun K. Kota and Ketul C. Popat**

Supporting Information

Interaction of Blood and Bacteria with Slippery Hydrophilic Surfaces

Prem Kantam, Vignesh K Manivasagam, Tarun Kumar Jammu, Roberta Maia Sabino, Sravanthi Vallabhuneni, Young Jae Kim, Arun K Kota and Ketul C Popat**

P. Kantam, V. K. Manivasagam, T. K. Jammu, K. C. Popat, A. K. Kota
Department of Mechanical Engineering,
Colorado State University,
Fort Collins CO 80523, USA
E-mail: ketul.popat@colostate.edu

R. M. Sabino, K. C. Popat
School of Advanced Materials Discovery,
Colorado State University,
Fort Collins CO 80523, USA

S. Vallabhuneni, Y. J. Kim, A. K. Kota
Department of Mechanical and Aerospace Engineering,
North Carolina State University,
Raleigh NC 27695, USA
E-mail: akota2@ncsu.edu

K. C. Popat
School of Biomedical Engineering,
Colorado State University,
Fort Collins, CO 80523, USA

Keywords: slippery, hydrophilic, platelet adhesion, platelet activation, bacterial adhesion

Section S1. Characterization of surface chemistry with XPS

We characterized the surface chemistry of untreated silicon wafers and SLIC surfaces using x-ray photoelectron spectroscopy (XPS). We analyzed the high resolution C1s spectra (Figure 1A in the main manuscript), O1s spectra (Figure S1A) and Si2p spectra (Figure S1B).

The high resolution C1s spectra of untreated silicon wafers showed C-C peak at 285 eV, which is due to adventitious carbon. The high resolution C1s spectra of SLIC surfaces showed an ether carbon peak at 286.5 eV, which is characteristic of PEG groups [1]. This indicates the presence of covalently bound PEG on SLIC surfaces, but not on untreated silicon wafers.

The high resolution O1s spectra of both untreated silicon wafers and SLIC surfaces showed peaks at about 533 eV. While the O1s peak for untreated silicon wafers is indicative of oxygen from the native oxide layer [2], the O1s peak for SLIC surfaces is indicative of ether oxygen of PEG groups [3].

The high resolution Si2p spectra of both untreated silicon wafers and SLIC surfaces showed two distinct peaks at about 100 eV and 103 eV. The Si peak at 100 eV is characteristic of elemental silicon [2b, 4]. The Si peak at 103 eV is characteristic of the Si-O species, which arise from native oxide layer for untreated silicon wafers [4a, 5], and PEG silanes covalently bound via the Si-O bond for SLIC surfaces [4b, 5a, 6].

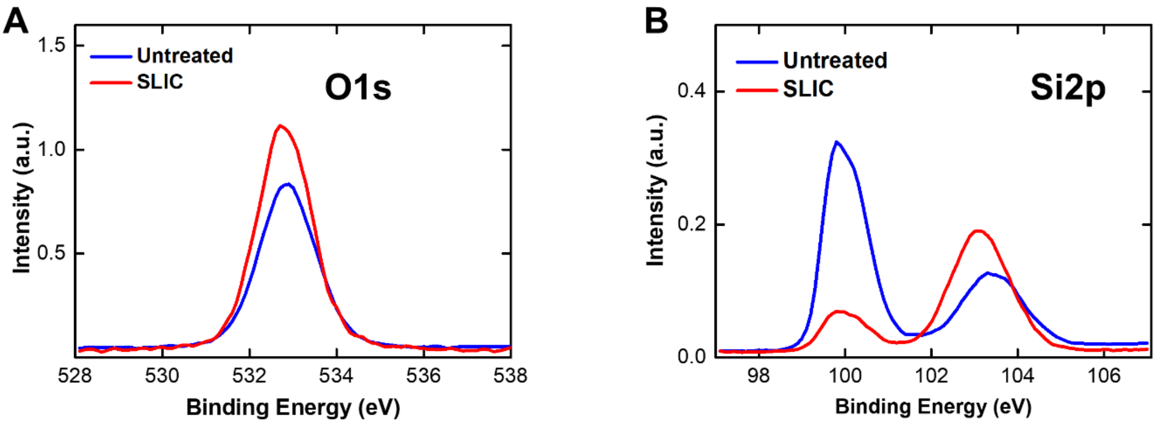

Figure S1. XPS spectra of untreated silicon and SLIC surfaces. (A) High resolution O1s spectra. (B) High resolution Si2p spectra.

Section S2. Characterization of contact angles

The contact angles of water indicate that both untreated silicon wafers and SLIC surfaces are hydrophilic. The contact angle hystereses of water indicate that SLIC surfaces are significantly more slippery compared to untreated silicon wafers (see Table S1).

Table S1. Contact angles and contact angle hystereses of water on untreated silicon wafers and SLIC surfaces.

	θ_{adv} (°)	θ_{rec} (°)	$\Delta\theta$ (°)
Untreated silicon	34°	17°	17°
SLIC surfaces	41°	37°	4°

Section S3. Durability of SLIC surfaces

We assessed the durability of our SLIC surfaces under extended exposure to air, water, steam and sliding water droplets through periodic measurements of advancing and receding contact angles. Our results indicated that SLIC surfaces maintained virtually unchanged advancing and receding contact angles (i.e., retained hydrophilicity and slipperiness) after exposure to air for 5 days (Figure S3A), immersion in water for 5 days (Figure S3B), exposure to steam at 100°C and atmospheric pressure for 48 hours (Figure S3C), and after sliding 50,000 water droplets across the surface (Figure S3D). While these results show the promise of SLIC surfaces, a more comprehensive and application-specific assessment of durability is necessary before practical utility.

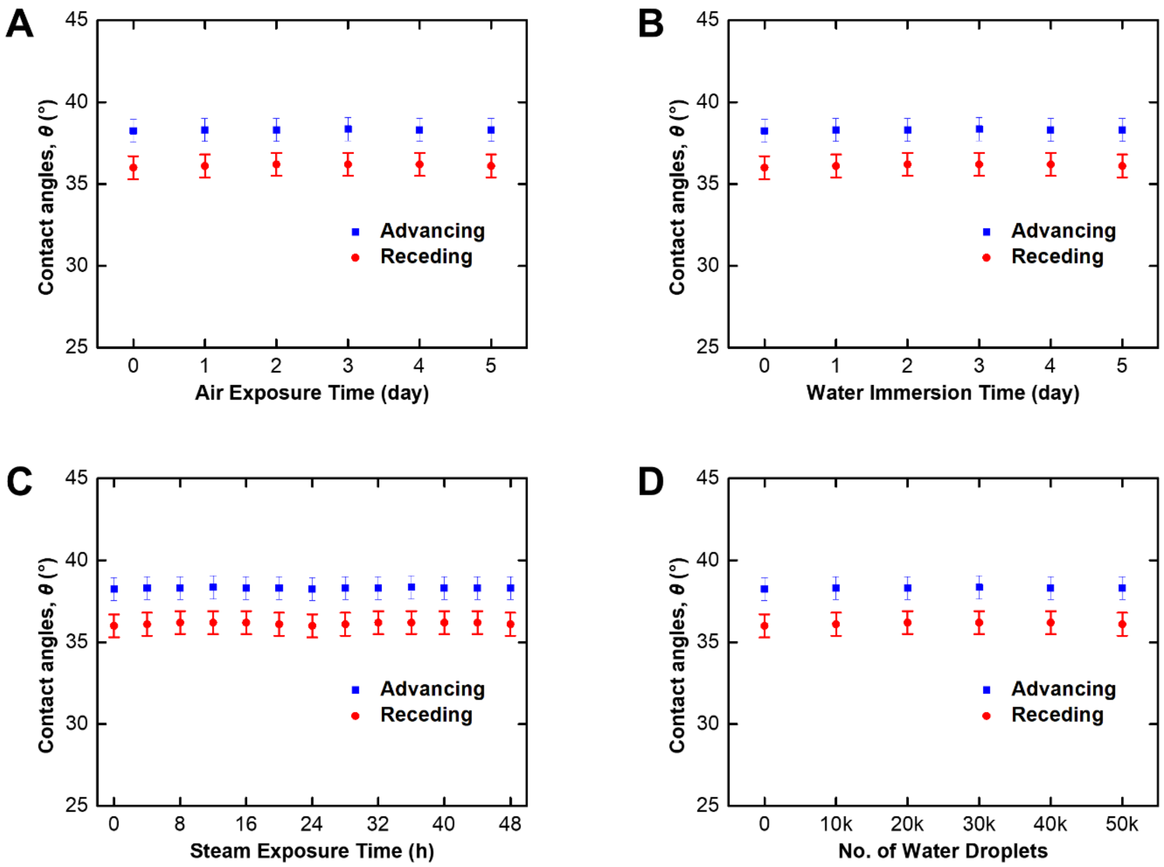

Figure S2. Durability of SLIC surfaces. (A), (B), (C) and (D) Advancing and receding contact angles of water on a SLIC surfaces as a function of air exposure time, water immersion time, steam exposure time and number of water droplets sliding past the surface, respectively.

References:

[1] a) S. Sharma, R. W. Johnson, T. A. Desai, *Biosensors and Bioelectronics* **2004**, 20, 227; b) S. Contarini, S. Howlett, C. Rizzo, B. De Angelis, *Applied surface science* **1991**, 51, 177; c) L. Britcher, T. J. Barnes, H. J. Griesser, C. A. Prestidge, *Langmuir* **2008**, 24, 7625.

[2] a) Y. Leprince-Wang, *Surface and Coatings Technology* **2002**, 150, 257; b) A. Alam, M. Howlader, M. Deen, *ECS Journal of Solid State Science and Technology* **2013**, 2, P515; c) Z. Sun, K. Sun, H. Zhang, H. Liu, D. Wu, X. Wang, *Solar Energy Materials and Solar Cells* **2021**, 225, 111069.

[3] a) M. Cerruti, S. Fissolo, C. Carraro, C. Ricciardi, A. Majumdar, R. Maboudian, *Langmuir* **2008**, 24, 10646; b) C. Tang, L. Feller, P. Rossbach, B. Keller, J. Vörös, S. Tosatti,

M. Textor, *Surface science* **2006**, 600, 1510; c) A. Dougherty, C. Harper, F. Iskandar, I. Arif, G. Dougherty, *Journal of Science: Advanced Materials and Devices* **2018**, 3, 419.

[4] a) Y. Xu, K. Han, J. Xiang, X. Wang, *IEEE Access* **2020**, 8, 159162; b) D. Gallach, G. R. Sánchez, A. M. Noval, M. M. Silván, G. Ceccone, R. M. Palma, V. T. Costa, J. M. Duart, *Materials Science and Engineering: B* **2010**, 169, 123.

[5] a) S. Sharma, R. W. Johnson, T. A. Desai, *Applied Surface Science* **2003**, 206, 218; b) P. Dietrich, C. Streeck, S. Glamsch, C. Ehlert, A. Lippitz, A. Nutsch, N. Kulak, B. Beckhoff, W. Unger, *Analytical chemistry* **2015**, 87, 10117.

[6] M. Veiseh, M. Zhang, *Journal of the American Chemical Society* **2006**, 128, 1197.
